# Rising Above Falls: Resilience, Fear of Falling, and Impact on Functional and Psychosocial Outcomes

**DOI:** 10.1093/geroni/igaf122.981

**Published:** 2025-12-31

**Authors:** Rahul Malhotra, Lakkhina Troeung, Abhijit Visaria, Vanessa Koh, Kok Yang Tan, Wen Huey Lim, Angelique Chan

**Affiliations:** Duke-NUS Medical School, Singapore, Singapore; Duke-NUS Medical School, Singapore, Singapore; Duke-NUS Medical School, Singapore, Singapore; Duke-NUS Medical School, Singapore, Singapore; Duke-NUS Medical School, Singapore, Singapore; Duke-NUS Medical School, Singapore, Singapore; Duke-NUS Medical School, Singapore, Singapore

## Abstract

Falls are associated with multiple adverse outcomes for older adults including loss of independence, social isolation, and increased mortality risk. The fear of falling (FoF) is common among older adults and has been shown to significantly predict post-fall outcomes across numerous international studies. This study examines the proposed causal pathways between resilience, falls, FoF, and functional and psychosocial outcomes using a moderated-mediation approach. Structural equation modelling (SEM) using the TARGET cohort (n = 2,291) showed that self-reported fall count in the prior 12-months significantly predicted poorer functional independence (Modified Barthel Index; MBI, Instrumental Activities of Daily Living; IADL), greater depression (Patient Health Questionnaire-9; PHQ-9) and poorer quality of life (EuroQol-5D; EQ5D). FoF (Iconographical Falls Efficacy Scale) fully mediated the paths between falls and MBI, IADL and EQ5D, and partially mediated the path between falls and PHQ-9. Resilience (Brief Resilience Scale; BRS) significantly moderated the effects of falls on FoF, and FoF and all outcomes. The effect of falls on FoF was largest for the Low resilience (β = 4.52, p < 0.002) compared to Normal (β = 1.39, p < 0.000) and High (β = 1.20, p = 0.034) groups. Similarly, FoF had the largest indirect effects on outcomes in the Low resilience group, PHQ-9 (β = 0.46, p = 0.044), MBI (β=-2.34,p=0.006), IADL (β = 0.79,p=0.003), and EQ5D (β=-0.05,p=0.008). In contrast, FoF was not significantly predictive of all four outcomes in the High resilience group. Thus, resilience is an important protective mechanism for older adults. This presentation will discuss clinical implications, notably the importance of developing targeted “resilience-boosting” interventions to improve post-fall outcomes.

